# Transient EDTA‐dependent pseudothrombocytopenia and ulcerative colitis recurrence during chemotherapy: A case of misleading platelet count results attributable to a laboratory artifact

**DOI:** 10.1002/ccr3.8153

**Published:** 2023-11-21

**Authors:** Ayako Watanabe, Hirokazu Toshima, Misa Saeki, Takuya Nagata, Tatsuo Koyanagi, Masumi Minamizawa, Yuka Kashiwabara, Koji Kobayashi, Ken Shimada, Kenji Momo

**Affiliations:** ^1^ Department of Pharmacy Showa University Koto Toyosu Hospital Tokyo Japan; ^2^ Department of Hospital Pharmaceutics School of Pharmacy Showa University Tokyo Japan; ^3^ Department of Oncology Showa University Koto Toyosu Hospital Tokyo Japan; ^4^ Department of Clinical Genomics, BML, Inc Tokyo Japan

**Keywords:** autoimmune disease, delayed treatment, laboratory artifact, misleading diagnosis, transient EDTA‐dependent pseudothrombocytopenia

## Abstract

**Key Clinical Message:**

EDTA‐dependent pseudothrombocytopenia as well as myelosuppression should be suspected when thrombocytopenia occurs in patients with autoimmune disease during chemotherapy.

**Abstract:**

A patient with pancreatic cancer and ulcerative colitis developed transient ethylenediaminetetraacetic acid (EDTA)‐dependent pseudothrombocytopenia with exacerbation of ulcerative colitis during chemotherapy. Unfortunately, pseudothrombocytopenia could not be immediately detected because thrombocytopenia was masked by a reasonable time course of adverse events associated with chemotherapy and ulcerative colitis recurrence. When thrombocytopenia occurs during chemotherapy, especially in patients with autoimmune diseases, EDTA‐dependent pseudothrombocytopenia and bone marrow suppression caused by anti‐cancer agents should be suspected.

## Graphical abstract

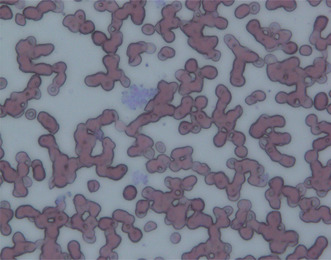

## 1 INTRODUCTION

Ethylenediaminetetraacetic acid (EDTA)‐dependent pseudothrombocytopenia is a laboratory artifact that occurs in approximately 0.1% of clinical cases.^1^, ^2^ Medical professionals often blindly rely on laboratory data that have a crucial role in determining treatment strategies. Pseudothrombocytopenia and, particularly, platelet count results can lead to the misdiagnosis of disseminated intravascular coagulation and heparin‐induced thrombocytopenia or delays in chemotherapy.^3^ Although most medical professionals are aware of EDTA‐dependent pseudothrombocytopenia, the determination of this condition in clinical practice is challenging, especially for cases involving acute phases, appropriate time courses, specific diseases, and medications preceding thrombocytopenia. We present a case of a patient with pancreatic cancer and autoimmune disease who experienced transient EDTA‐dependent pseudothrombocytopenia and worsening autoimmune disease while undergoing chemotherapy. This condition may have contributed to the delayed initiation of anti‐cancer therapy.

## 2 CASE PRESENTATION

**FIGURE 1:**
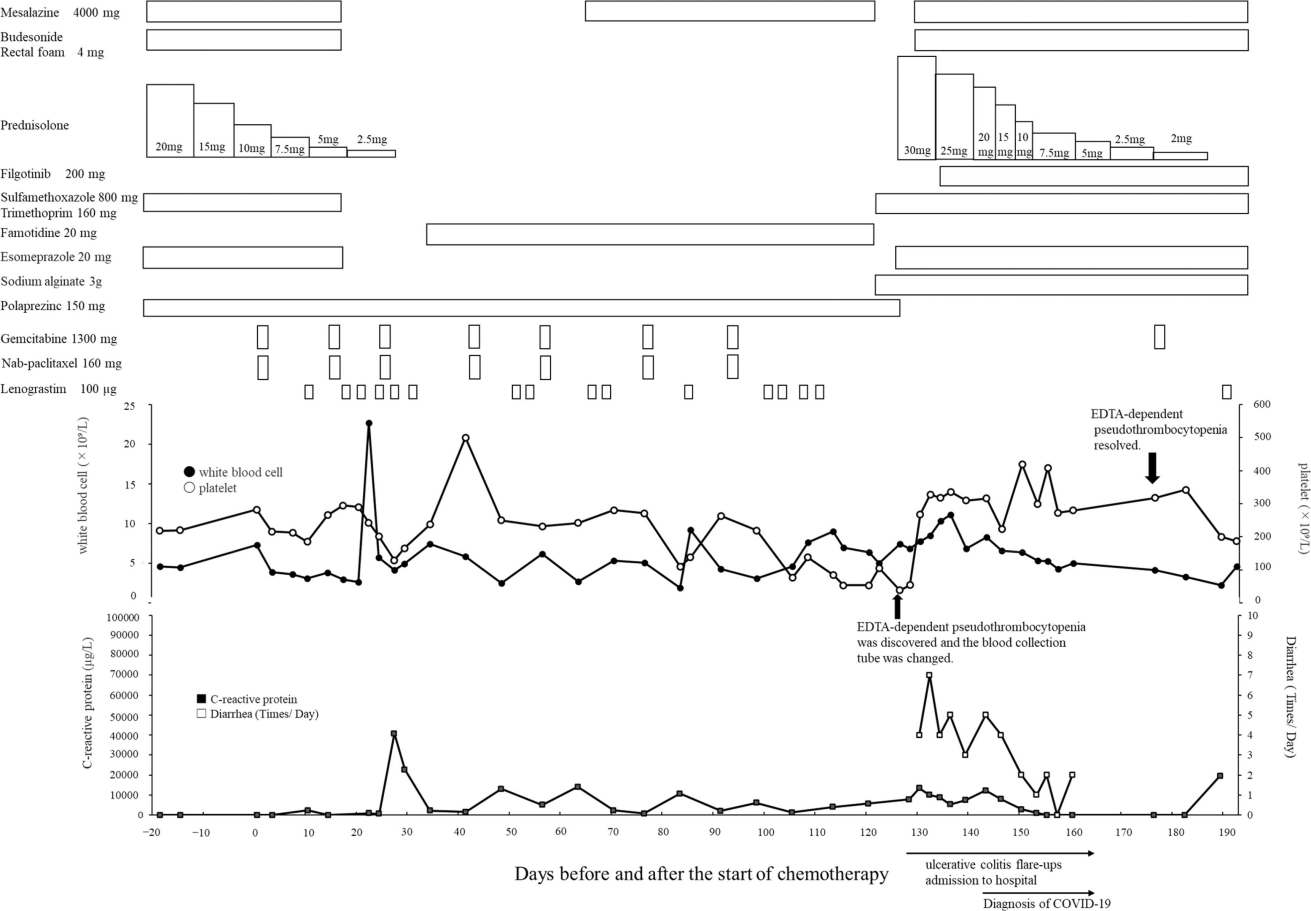
Clinical course of the present case.

A 54‐year‐old man with a history of a pituitary tumor (treated with surgery 27 years previously), lateral cervical cyst, and ulcerative colitis presented with unresectable pancreatic cancer involving the stomach that resulted in stomach stenosis. The patient's baseline characteristics included the following: weight, 69.1 kg; height, 170 cm; and body surface area, 1.807 m^2^. Ulcerative colitis remission after treatment with prednisolone (40 mg/day), mesalazine (4000 mg/day), and budesonide rectal foam (4 mg/day) was confirmed before initiating chemotherapy (Figure 1).

The chemotherapy regimen comprised nanoparticle albumin‐bound paclitaxel (160 mg, calculated based on 125 mg/m^2^ with an 80% dose) and gemcitabine (1300 mg, calculated based on 1000 mg/m^2^ with an 80% dose) on Days 1, 8, and 15 during a 28‐day cycle. Before the third course of chemotherapy (Days 1–90), the platelet count generally fluctuated within the normal range (150–450 × 10^9^/L), and neutropenia was treated with granulocyte colony‐stimulating factor. During the third course of chemotherapy (after Day 105), his platelet count decreased to Common Terminology Criteria for Adverse Events grade 3 (25–50 × 10^9^/L). Additionally, the patient experienced Grade 2 diarrhea. We considered his thrombocytopenia to be caused by chemotherapy and his diarrhea to be caused by ulcerative colitis recurrence. Prednisolone (40 mg/day), mesalazine (4000 mg/day), budesonide rectal foam (4 mg/day), sulfamethoxazole (800 mg), trimethoprim (160 mg/day), polaprezinc (150 mg/day), and esomeprazole (20 mg/day) were administered.

### 2.1 Platelet count changes and pseudothrombocytopenia diagnosis

Initially, we suspected that thrombocytopenia was caused by chemotherapy, particularly gemcitabine. However, upon further investigation, the physician noted that fluctuations in the platelet count that occurred during Days 105–126 were not consistent with the expected clinical course of chemotherapy. This was evident from the drastic independent increases and decreases in the platelet count that were not correlated with the administration of anti‐cancer agents on Day 91.

Additionally, the patient experienced severe diarrhea that was attributed to ulcerative colitis recurrence. Therefore, we attempted to control his ulcerative colitis to enable the continuation of anti‐cancer therapy and investigate the significant changes in the platelet count.

**FIGURE 2:**
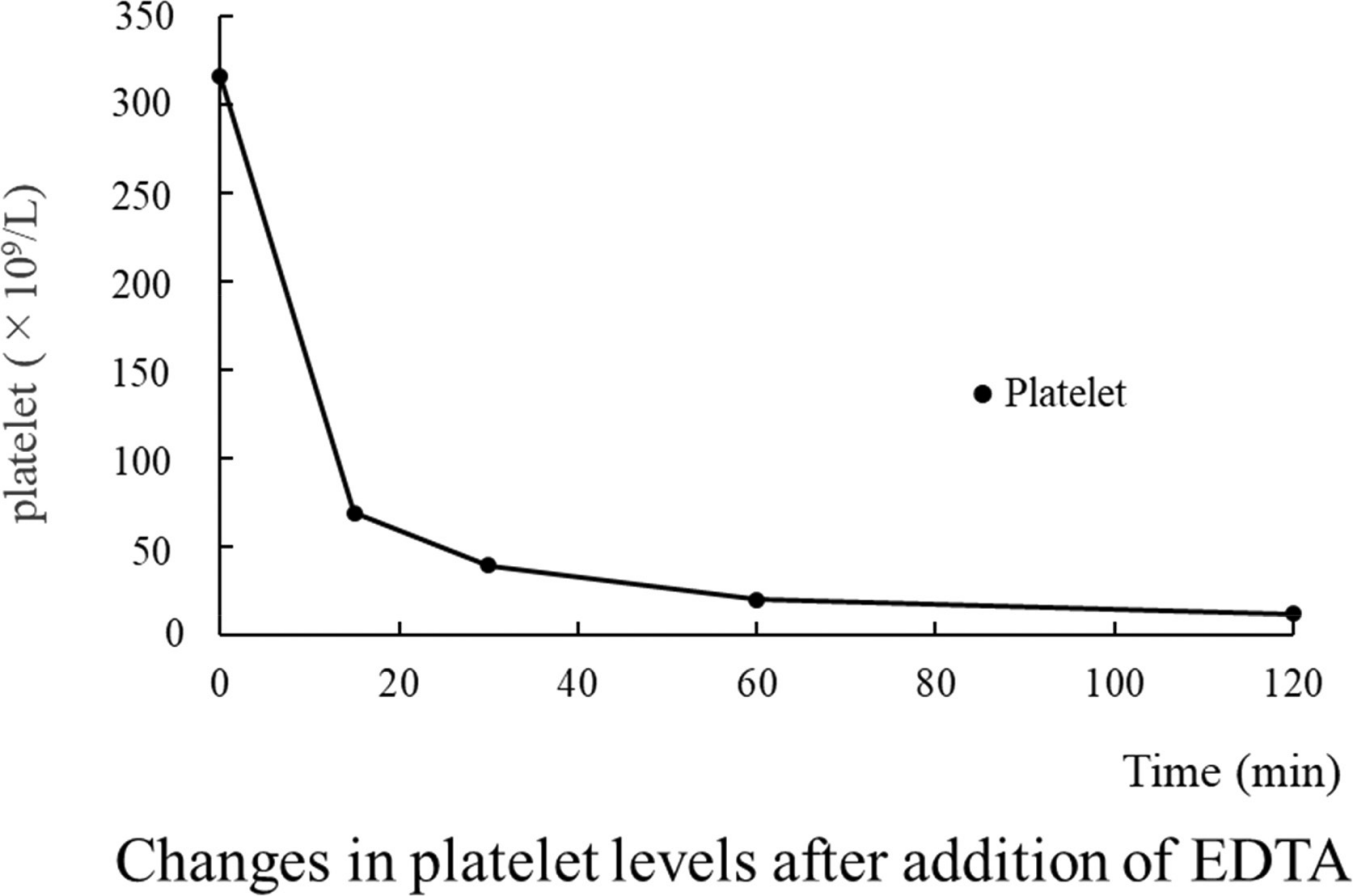
Changes in the platelet count after the addition of ethylenediaminetetraacetic acid (EDTA).

**FIGURE 3:**
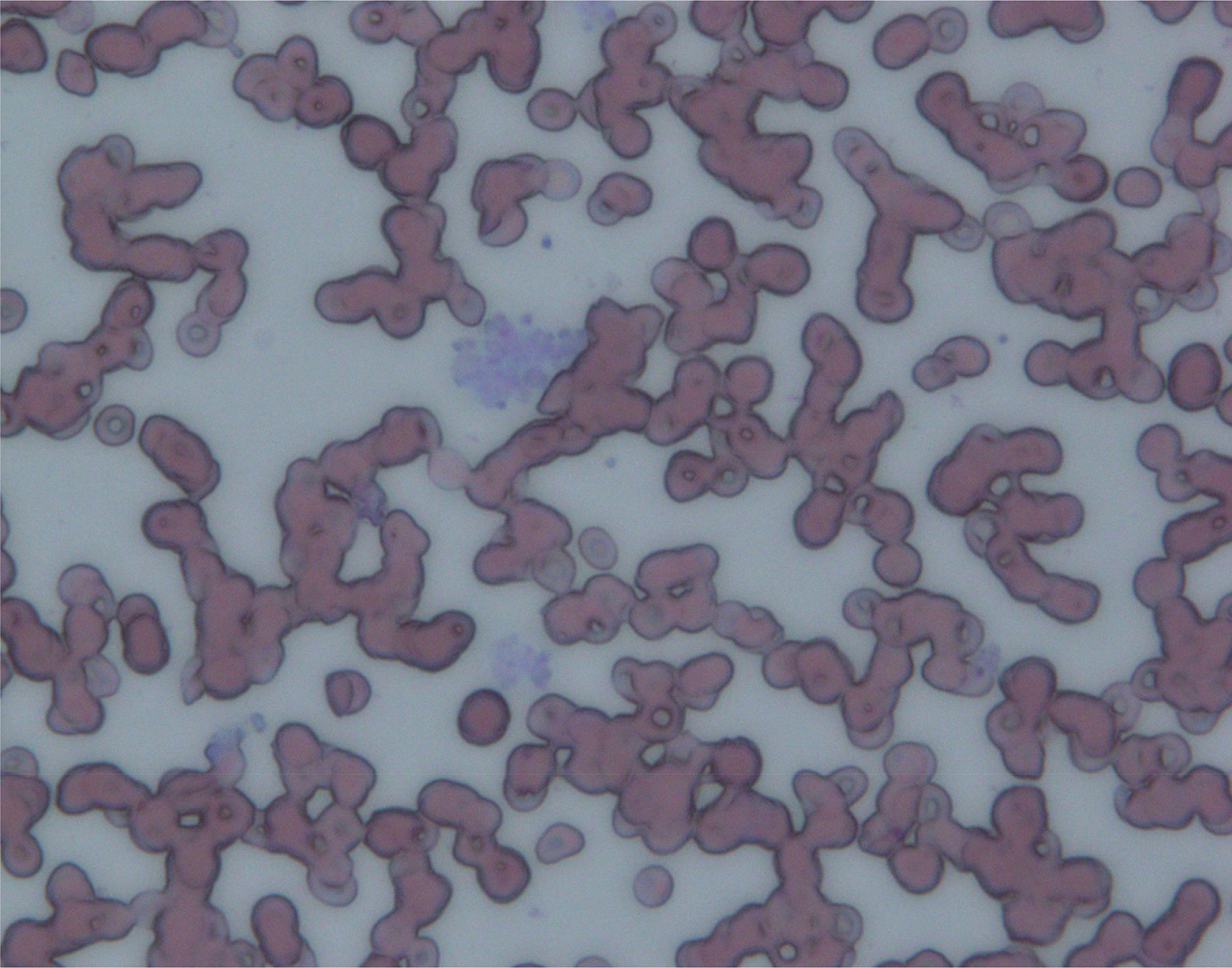
A peripheral smear during thrombocytopenia.

Fortunately, his ulcerative colitis improved after several weeks of treatment. Subsequently, because of the observed changes, we measured the platelet count over time using EDTA blood collection tubes, which are commonly used to measure the blood count. The results revealed a decreasing trend in the platelet count, which was correlated with the retention time in the EDTA blood collection tube (Figure 2). However, this phenomenon was not observed when heparin‐containing blood collection tubes were used. Additionally, platelet aggregation was observed in the peripheral smear (Figure 3). This aggregation pattern indicated the occurrence of pseudothrombocytopenia and led to the diagnosis of EDTA‐dependent pseudothrombocytopenia on Day 126. Thereafter, EDTA‐free blood collection tubes were used to measure the blood count. No significant changes in the platelet count or blood count measurements using EDTA blood collection tubes were observed when chemotherapy was restarted with gemcitabine alone after ulcerative colitis remission. Consequently, we revised the diagnosis to include transient EDTA‐dependent pseudothrombocytopenia.

Following the remission of ulcerative colitis, the patient contracted COVID‐19 between Days 143 and 160. Subsequent to recovery, the patient was discharged. Importantly, no notable fluctuations in platelet counts were observed throughout the course of the COVID‐19 illness. On Day 176, we resumed the fourth course of chemotherapy with co‐administered medicines for ulcerative colitis. No significant changes in the platelet count were observed after the fourth course (Figure 1).

## 3 DISCUSSION

We encountered a patient with cancer and ulcerative colitis who developed pseudothrombocytopenia. Thrombocytopenia, defined as a platelet count less than 150 × 10^9^/L, can be caused by various factors, such as viral infections, malignancy, chemotherapy, and autoimmune diseases. Thrombocytopenia related to chemotherapy is a common occurrence that affects approximately 13% of patients undergoing treatment for solid tumors.^4^ In contrast, EDTA‐dependent pseudothrombocytopenia is a rare occurrence caused by a laboratory artifact in approximately 0.1% of cases.^1^, ^2^ Pseudothrombocytopenia is characterized by normal platelet counts in vivo but decreased platelet counts when blood is collected in EDTA‐containing tubes. One of the mechanisms underlying pseudothrombocytopenia is the chelation of divalent cations by EDTA, which alters the epitope of the platelet membrane glycoprotein IIb/IIIa, thus leading to immunoglobulin reactions in the blood.^5^ Kocum et al. reported that this phenomenon can result in misdiagnoses and mismanagement in clinical settings, especially for patients who are unable to receive reperfusion therapy during acute myocardial infarction.^6^ EDTA‐dependent pseudothrombocytopenia resulted in delayed anti‐cancer drug administration for our patient with pancreatic cancer.

We observed transient EDTA‐dependent pseudothrombocytopenia concurrent with ulcerative colitis recurrence in our patient. Drug‐induced pseudothrombocytopenia associated with EDTA can occur with medications such as valproic acid, olanzapine, and sunitinib.^7^, ^8^, ^9^ It is also associated with underlying diseases, such as autoimmune disorders, neoplastic diseases, atherosclerosis‐related conditions, liver disease, and infections.^10^, ^11^, ^12^, ^13^, ^14^, ^15^, ^16^, ^17^ Furthermore, a transient association with viral infections has been suggested.^18^, ^19^ However, conflicting study results have indicated that it may not be associated with any underlying disease.^20^, ^21^ The mechanism and cause of EDTA‐dependent pseudothrombocytopenia are not fully understood. Nonetheless, it is crucial for medical professionals to be aware of this condition and consider it as a potential danger, particularly when encountering patients with abnormal platelet counts during the natural clinical course.

## 4 CONCLUSION

In conclusion, we encountered a case of EDTA‐dependent thrombocytopenia in a patient undergoing anti‐cancer therapy. This phenomenon can result in unnecessary treatment or delayed necessary treatment, thereby placing patients at risk for serious complications. It is necessary to perform careful monitoring and consider the possibility of EDTA‐dependent pseudothrombocytopenia when unusual changes in the platelet count occur.

## AUTHOR CONTRIBUTIONS

**Ayako Watanabe:** Investigation; writing – original draft. **Hirokazu Toshima:** Project administration; validation. **Misa Saeki:** Investigation. **Takuya Nagata:** Investigation. **Tatsuo Koyanagi:** Validation; visualization. **Masumi Minamizawa:** Validation; visualization. **Yuka Kashiwabara:** Supervision; validation. **Koji Kobayashi:** Validation. **Ken Shimada:** Supervision; validation. **Kenji Momo:** Project administration; supervision; writing – original draft.

## FUNDING INFORMATION

None.

## CONFLICT OF INTEREST STATEMENT

The Department of Hospital Pharmaceutics, School of Pharmacy, Showa University received funds from Ono for a contract research project according to a collaborative research agreement. Ken Shimada is medical adviser of Ono, Taiho, and Sawai. As a potential conflict of interest, Hospital Pharmaceutics received research grants from Daiichi Sankyo, Mochida, Shionogi, Ono, Taiho, Nippon–Kayaku, and Bayer. Takuya Nagata received an honorarium fee for presentations from Sanofi. Kenji Momo received an honorarium fee for presentations from Nippon–Kayaku, Eisai and Abbvie. Yuka Kashiwabara received an honorarium fee for presentations from Daiichi Sankyo. Hirokazu Toshima received an honorarium fee for presentations from Daiichi Sankyo, Taiho, Bristol–Myers. Ken Shimada received an honorarium fee for presentations from Daiichi Sankyo, Ono, Taiho, Yakult, Bristol–Myers. The other authors declare no conflict of interest associated with this manuscript.

## CONSENT

We obtained written informed consent from the patient for publication of this report.

## Data Availability

All information in this case is included in this published article.
